# Objectively measured the impact of ambient air pollution on physical activity for older adults

**DOI:** 10.1186/s12889-024-18279-2

**Published:** 2024-03-15

**Authors:** Jiali Cheng, Yin Wu, Xiaoxin Wang, Hongjun Yu

**Affiliations:** 1 Faculty of Public Physical Education, Hebei Normal University, 050024 Shijiazhuang, China; 2 The Experimental High School Attached to Beijing Normal University, 100032 Beijing, China; 3 Department of Physical Education, Tsinghua University, Tsinghua Yuan Str, 100084 Beijing, China

**Keywords:** Air pollution, Physical activity, Older adults, Objectively measured

## Abstract

**Background:**

Air pollution poses a significant health risk to the human population, especially for vulnerable groups such as the elderly, potentially discouraging their engagement in physical activity. However, there is a lack of sufficient objective and longitudinal data in current research on how air pollution affects physical activity among older adults. With these gaps, we aimed to explore the relationship between air pollution and objective measurement-based physical activity among older adults by engaging in a longitudinal study design.

**Methods:**

A total of 184 older adults were recruited from three cities with varying levels of air quality. Mean daily minutes of physical activity were measured with 7 consecutive days of accelerometer monitoring (ActiGraph GT3X-BT). Corresponding air pollution data including daily PM_2.5_ (µg/m^3^), PM_10_ (µg/m^3^) and air quality index (AQI) were sourced from the China National Environmental Monitoring Centre at monitor locations close to older adults’ addresses. Associations between air quality and physical activity were estimated using a fixed effect model, adjusting for average daytime temperature, rain, age and weight.

**Results:**

AQI and PM_2.5_ were observed to exhibit significant, inverse, and linear associations with mean daily walk steps, minutes of light physical activity (LPA), moderate physical activity (MPA) and moderate-to-vigorous physical activity (MVPA) in the single variable models. A one-level increase in AQI corresponded to a decline in 550.04 steps (95% [CI] = -858.97, -241.10; *p* < 0.001), 10.43 min (95% [CI] = -17.07, -3.79; *p* < 0.001), 4.03 min (95% [CI] = -7.48, -0.59; *p* < 0.001) and 4.16 min (95% [CI] = -7.77, -0.56; *p* < 0.001) in daily walking steps, LPA, MPA, and MVPA, respectively. A one-level increase in PM_2.5_ correlated with a decline in daily walk steps, LPA, MPA and MVPA by 361.85 steps (95% [CI] = -516.53, -207.16; *p* < 0.001), 8.97 min (95% [CI] = -12.28, -5.66; *p* < 0.001), 3.73 min (95% [CI] = -5.46, -2.01; *p* < 0.001,) and 3.79 min (95% [CI] = -5.59, -1.98; *p* < 0.001), respectively. However, PM_10_ displayed a significant negative association exclusively with LPA, with one-level increase in PM_10_ resulting in a 3.7-minute reduction in LPA (95% [CI] = -6.81, -0.59, *p* < 0.05).

**Conclusion:**

Air pollution demonstrates an inverse association with physical activity levels among older adults, potentially discouraging their engagement in physical activity. Different air quality indicators may exert varying impacts on physical activity. Future studies are warranted to enhance policy interventions aimed at reducing air pollution and promoting physical activity.

## Introduction

Air pollution stands as a prominent global environmental health issue [1], resulting in the exacerbation of various diseases, including but not limited to decreased lung function, stroke, myocardial infarction, hypertension, asthma, bronchitis and premature mortality [2–5], especially for vulnerable groups such as the elderly [6, 7]. Some studies indicated that air pollution is associated with reduced lung function, elevated hypertension, and diverse respiratory and cardiovascular symptoms [2, 3], consequently impacting exercise capacity [8–10]. Despite observed declines in air pollutant levels over recent decades, the issue of poor air quality in developing countries remains a substantial public health concern [11, 12]. A study that gathered hourly air pollution data from more than 1500 monitoring stations across 32 provinces in China revealed that 92% of China’s residents encountered over 120 h of unfavorable air conditions annually [11].

Physical activity exerts an essential role in human health benefits, especially for older adults [13, 14]. Compelling evidence reveals that physical activity not only effectively reduces the risk of cardiovascular disease, obesity, stroke, type-II diabetes, osteoporosis, colon cancer and other diseases associated with air pollution among the elderly, but also enhances their mental health [15, 16]. Additionally, physical activity has been shown to increase their lifespan and healthy life expectancy [17, 18]. Research evidence has also suggested a “dose-response” connection between health status and the intensity of physical activity, indicating that engaging in higher intensity physical activities leads to greater health benefits [19–25]. Therefore, it is of paramount importance to enhance physical activity levels.

Current literature contains studies exploring air pollution’s impact on health behavior. Air pollution alerts can reduce fragile groups’, such as the elderly’s, outdoor activities [26, 27]. For example, warnings and alerts issued by local air pollution monitoring government institutions can reduce outdoor cycling behavior by 14–35% [28]. A study also showed that individuals mitigate pollution exposure by reducing the duration of outdoor vigorous physical activity (VPA), averaging an 18-minute reduction on days with air quality alerts [29]. Furthermore, studies have indicated varying degrees of air pollution’s impact on physical activity levels [30–35]. For example, a study from Korea reported both PM_10_ and PM_2.5_ levels presented significant negative impacts on the distances and durations of outdoor biking [36]. A meta-analysis drawing from various cross-sectional researches concluded that each unit (µg/m^3^) increase in ambient PM_2.5_ levels resulted in a 10% higher chance of physical inactivity [4]. Moreover, a merger of data from a South Korea study showed that the duration of walking decreased as the PM_10_ increased [37]. A previous mixed-age study exploring the correlation between physical activity and air quality index (AQI) showed that more than half of the participants adjusted the timing or frequency of their physical exercise in response to the AQI levels in their neighborhood [38]. A longitudinal cohort study of adults aged 18 years or older from California revealed that poor air quality was significantly linked to a reduction in accelerometer-based daily step counts. Specifically, when the AQI surpassed 200, step counts decreased by 18% compared to levels below 100 [39].

Although the aforementioned works have been done, three notable gaps persist in the scientific literature. First, there is a lack of studies using objective measurements to evaluate physical activity among older adults, all studies have relied on self-reported physical activity data [4, 31, 32, 40]. Second, while some studies have reported relationships between PM_2.5_, PM_10_ and physical activity levels of the elderly [9, 41], and between AQI and physical activity among mixed-age groups [38], no study has investigated the relationship between AQI and physical activity among older adults. Third, few studies have employed a longitudinal study design to explore how air pollution impacts physical activity, with most studies adopting a cross-section design [4, 32, 33, 40]. Even when longitudinal research was conducted, the primary focus was on diseased populations [42], non-elderly populations [35], or relied on self-reported methods to measure physical activity [34]. Hence, the objective of the study was to investigate how air pollution affects physical activity among older adults engaging objective measurements longitudinal study design.

## Methods

### Participants

The study recruited 184 participants from 46 communities in Beijing, Shijiazhuang, Hebei Province and Qinzhou, Guangxi Province during several periods (Beijing *N* = 116, Jan 11–18, 2018; Sep 28–Oct 11, 2019; Apr 16–27, 2021; May 6–17, 2021; Qinzhou *N* = 20, June 8–21, 2021; Shijiazhuang *N* = 48, July 14–23, 2021; July 23–31, 2021). Three cities present different air environments. According to a report from the Ministry of Ecology and Environment, Shijiazhuang is ranked in the bottom 20 nationwide regarding air quality, whereas Qinzhou is situated in the top 20, and Beijing falls in the middle range. All participants were healthy without diseases that could affect physical activity and were retired individuals aged 60 years or older. Recruitment information was disseminated through the Community Committee Office prior to this study. Individuals who were interested in our study provided written informed consent and completed a paper-pencil demographical information questionnaire including age, gender, weight and height. During each measurement period, participants were told to fasten accelerometers on their non-dominant waist. Air pollution data for the corresponding periods were collected from China National Environmental Monitoring Centre. Four participants who did not complete measurements were excluded because of three devices without data and one device with invalid data (wearing duration less than three days). Consequently, 180 participants with valid data were included in our study. Approval for the study was granted by Tsinghua University Institutional Review Board (IRB 20110170).

### Air pollution exposure

We obtained air pollution data including hourly PM_2.5,_ PM_10_, and AQI from the China National Environmental Monitoring Centre. AQI is an air quality index, comprising five components: carbon monoxide, ozone, particle pollution (PM), nitrogen dioxide, and sulfur dioxide. One of the PM components is classified into PM_2.5_ and PM_10_ on the basis of particle diameter, which are defined as < 2.5 µg and < 10 µg respectively (available from: https://www.epa.gov/criteria-air-pollutants/naaqs-table).

**Fig. 1:**
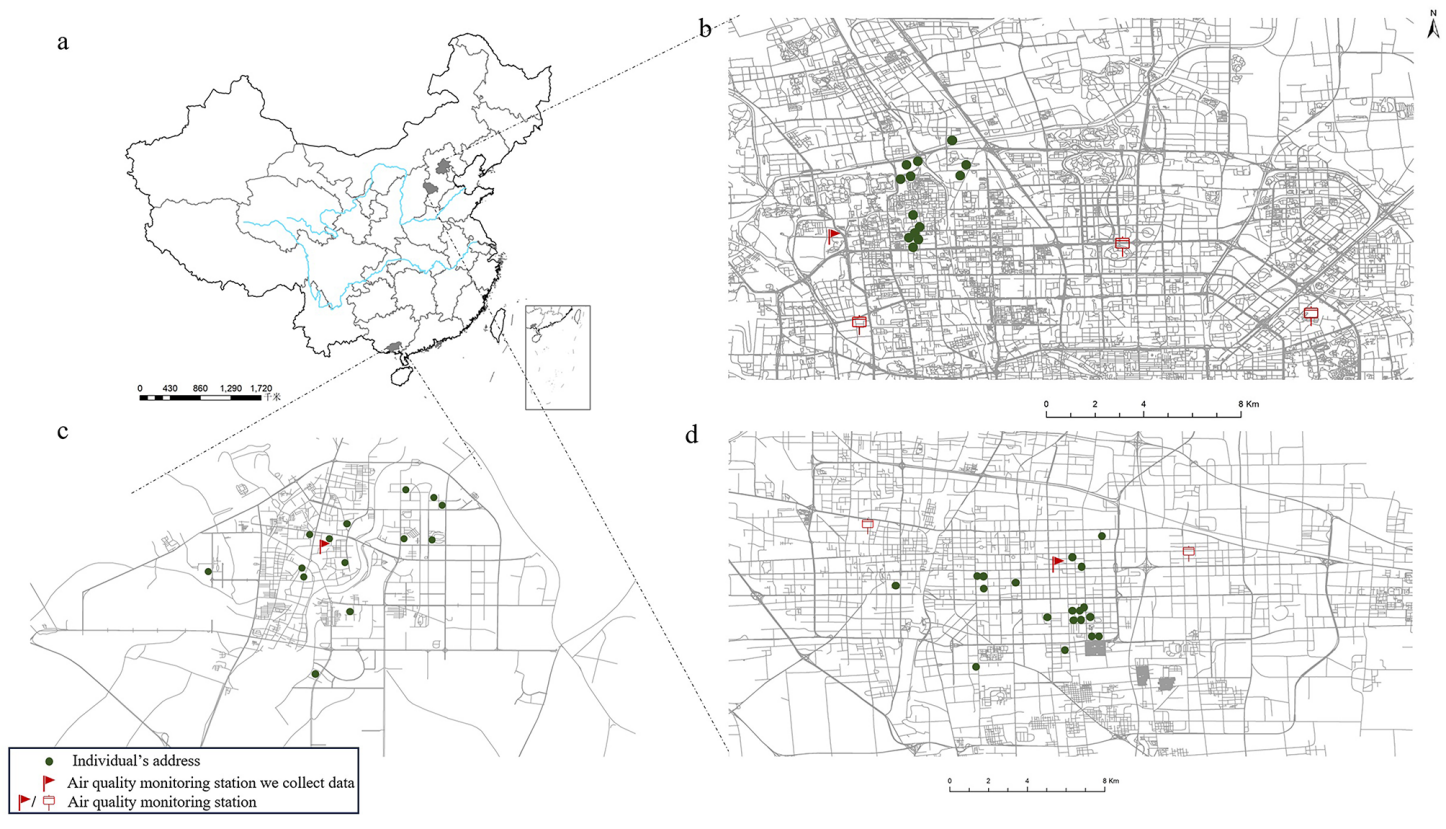
Map of air quality monitoring stations and individual’s addresses. *Notes*: **a**: Map of China; **b**: Map of Beijing; **c**: Map of Qinzhou; **d**: Map of Shijiazhuang; Map shows the locations of individual’s residential address and surrounding air quality monitoring sites

We selected monitoring sites closest to individuals’ residential addresses in the three cities for data collection (Fig. 1). These sites are Wanliu of the Haidian district, Beijing (air quality monitoring station ID:1007A), Century Park, Shijiazhuang (air quality monitoring station ID:1033A) and Qinzhou Environmental Monitoring Center, Qinzhou (air quality monitoring station ID:2502A), with an average distance of 4.71 km from the individual’s residential addresses. The AQI value ranges from 0 to 500 and is divided into six levels: Good AQI (0–50), Moderate AQI (51–100), Unhealthy for sensitive groups AQI (101–150), Unhealthy AQI (151–200), Very unhealthy AQI (201–300), Hazardous AQI (> 300). Similar to AQI, PM_2.5_ and PM_10_ are also categorized into six levels: very good, good, moderate, unhealthy for sensitive, unhealthy and very unhealthy.

### Physical activity measurement

We collected physical activity data concurrently with air pollution data. Physical Activity data were measured using an accelerometer (ActiGraph GT3X-BT), which is a reliable and valid objective measure [43]. The volunteers were asked to wear GT3X-BT accelerometer for over 7 continuous days, for at least 10 h a day. The monitors’ recording epoch was set for collecting data at 60 Hz. Individuals were told to remove the accelerometer only during showering, swimming, or other water-related activities. Data included in the analysis required a minimum wearing duration of at least three days, with ten hours of wear each day. We processed the walking steps, kcals in energy expenditure, metabolic equivalents (METs), daily light physical activity (LPA), VPA, very vigorous physical activity (VVPA), moderate-to-vigorous physical activity (MVPA) and sedentary behavior using ActiLife software (version 6.13.3). The physical activity intensity in all data is graded based on the Counts Per Minute (CPM), which is divided using the Freedon’s classical accelerometer model: sedentary behavior (0-99CPM), LPA (100–1951 CPM), MPA (1952–5724 CPM), MVPA (1952–9498 CPM), VPA (5725–9498 CPM) and VVPA (9499–16,000 CPM) [44].

### Statistical analysis

Statistical analysis was undertaken utilizing Stata 25.0. Descriptive statistics, including means, standard deviation (SD), and percentages of the 180 samples were calculated. Conducting a T-test was for evaluating whether the differences between the characteristics of male and female were significant. When *p* < 0.05, it was considered to be statistically significant. Fixed effect models adjusted for average daytime temperature, rain, age and weight were utilized for examining how air pollution, including PM_2.5_, PM_10_, and AQI, was related to physical activity variables.

## Results

### Participant characteristics

Table 1 displays the demographic characteristics of the 180 participants. Females made up nearly two-thirds (63.9%) of the participants. All participants wore GT3X-BT accelerometer for an average of 6.43 continuous days, totaling 1157days. Compliance with wearing the accelerometers was 5.85 days (SD = 1.46) for male and 6.76 (SD = 3.10) for female. The mean age, weight and BMI were 70.31 years old, 63.82 kg and 24.11 kg/m^2^ respectively. Additionally, male participants exhibited significantly greater height than their female counterparts (*p* < 0.05).

**Table 1:** The characteristics of subjects

Characteristics	Male	Female	Total	*p*
Gender, n (%)	65 (36.1)	115 (63.9)	180	
Compliance, total days	380 (32.84)	777 (67.16)	1,157	
Compliance days, mean (SD)	5.85 (1.46)	6.76 (3.10)	6.43 (2.66)	< 0.001
Age (yr), mean (SD)	71.85 (6.80)	69.43 (8.01)	70.31 (7.67)	0.097
Height (cm), mean (SD)	170.27 (5.26)	159.23 (5.83)	164.21 (7.83)	0.040
Weight (kg), mean (SD)	70.38 (9.83)	60.11 (7.67)	63.82 (9.82)	0.614
Body mass index(kg/m^2^), mean (SD)	24.34 (2.85)	23.92 (2.87)	24.11 (2.86)	0.490

### The air pollution variations

**Fig. 2:**
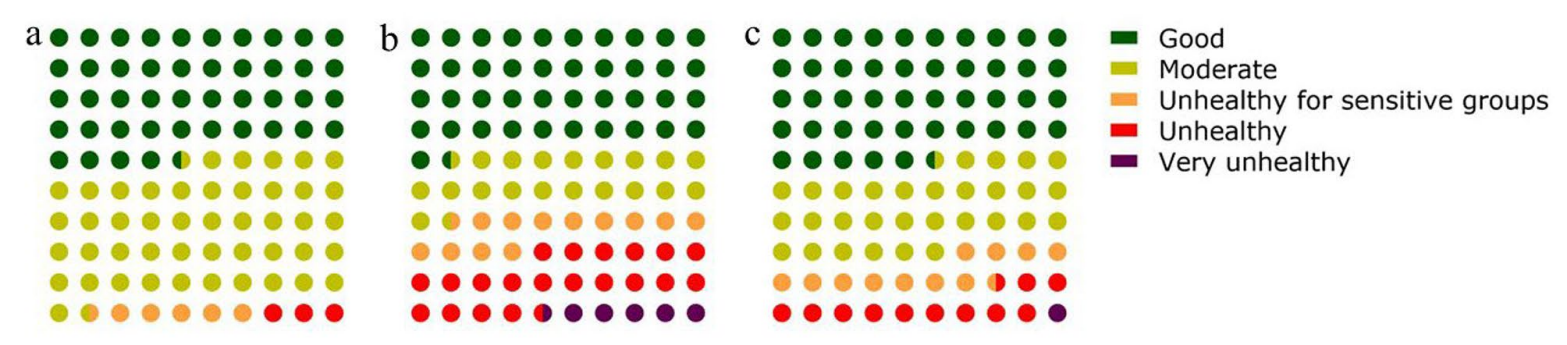
Dot plot of AQI (**a**), PM_2.5_ (**b**) and PM_10_ (**c**) percentage during the study period

Table 2 and Fig. 2 illustrate variations in air pollution measurements throughout the research period. There were 44.68%, 46.85%, 5.7% and 2.77% average AQI values at “Good” AQI (0–50), “Moderate” AQI (51–100), “Unhealthy for sensitive groups” AQI (101–150), “Unhealthy” AQI (151–200), respectively. Both the average daily PM_2.5_ and PM_10_ were at “Good” levels for over 40% of the study period.

**Table 2:** AQI, PM_2.5_ and PM_10_ categories

	Level	Alarm	Levels of health concern	Value range	N	%
AQI	1	Green	Good	0–50	517	44.68
	2	Yellow	Moderate	51–100	542	46.85
	3	Orange	Unhealthy for sensitive groups	101–150	66	5.70
	4	Red	Unhealth	151–200	32	2.77
	5	Purple	Very unhealthy	201–300	0	0
	6	Maroon	Hazardous	> 300	0	0
	1	Green	Very good^*^	1–15	245	21.18
**PM**_**2.5**_ (µg/m^3^)	2	Green	Good	15.1–25	237	20.48
	3	Yellow	Moderate	25.1–37.5	227	19.62
	4	Orange	Unhealthy for sensitive groups	37.6–50	149	12.88
	5	Red	Unhealth	50.1–75	234	20.22
	6	Purple	Very unhealthy	75.1–150	65	5.62
	1	Green	Very good^*^	11–45	525	45.38
	2	Green	Good	45.1–50	0	0
**PM**_**10**_ (µg/m^3^)	3	Yellow	Moderate	50.1–75	357	30.86
	4	Orange	Unhealthy for sensitive groups	75.1–100	128	11.06
	5	Red	Unhealth	100.1–150	133	11.50
	6	Purple	Very unhealthy	150.1–200	14	1.21
**Temperature** (°C)				-10–0	195	16.85
				0.1–10	2	0.17
				10.1–20	399	34.49
				20.1–30	542	46.85
				> 30	19	1.64
**Rain**	Yes				283	24.46
	No				874	75.54

*Notes*: Levels of health concern corresponding to Health Implications, (Very) Good: Satisfactory air quality with minimal or no risk from air pollution; Moderate: Acceptable air quality, though some pollutants may pose a moderate health risk for a very few individuals highly sensitive to pollution; Unhealthy for Sensitive Groups: Health impacts may affect susceptible groups, while the general public is expected to remain unaffected; Unhealthy: Noticeable health effects for the general population, with more serious effects for sensitive groups; Very Unhealthy: Health warnings indicating urgent circumstances, with the entire population at a higher risk of being affected; Hazardous: A health alert with the potential for more serious health effects across the population

^*^Very Good: WHO recommended short-term (24-hour) air quality guidelines (AQG) level before 2021; Good: After 2021

### The physical activity variations

Table 3 presents descriptive statistics for physical activity. The mean daily steps were 6663.97 steps. Participants on average engaged daily LPA, MPA, VPA, VVPA, MVPA and sedentary behavior was 159.22 (SD = 107.04), 59.26 (SD = 68.80), 1.59 (SD = 6.18), 0.64 (SD = 4.21), 61.49 (SD = 73.36) and 587.59 (SD = 279.42) minutes, respectively. The mean daily kcals and score of METs were 230.51 and 1.25.

**Table 3:** Average physical activity and sedentary behavior (*N* = 180)

Dependent variables	Mean (SD)
Steps of walk (daily steps)	6663.97 (4502.25)
LPA (daily minutes)	159.22 (107.04)
MPA (daily minutes)	59.26 (68.80)
MVPA (daily minutes)	61.49 (73.36)
VPA (daily minutes)	1.59 (6.18)
VVPA (daily minutes)	0.64 (4.21)
Kcals (daily kcals)	230.51 (308.12)
METs	1.25 (0.29)
Sedentary behavior (daily minutes)	587.59 (279.42)

*Notes*: LPA: light physical activity, MPA: moderate physical activity, MVPA: moderate-to-vigorous physical activity, VPA: vigorous physical activity, VVPA: very vigorous physical activity, METs: metabolic equivalent (s)

### The relationship between air pollution and physical activity

Table 4 illustrates the estimated impacts of AQI, PM_2.5_ and PM_10_ on individual-level daily physical activity through the fixed-effect model. Our findings indicate a converse correlation between air pollution variables and various aspects of daily physical activity, including total steps walked per day, total minutes of daily LPA, MPA and MVPA.

**Table 4:** Estimated impacts of AQI, PM_2.5,_ and PM_10_ on individual physical activity results

	Dependent variable	Total	
		Coefficient (95% CI)	# Observations (# participants)
**AQI**			
	Total steps of daily walk	-550.04*** (-858.97, -241.10)	1,157 (180)
	Total minutes of daily light physical activity	-10.43** (-17.07, -3.79)	1,157 (180)
	Total minutes of daily moderate physical activity	-4.03* (-7.48, -0.59)	1,157 (180)
	Total minutes of daily MVPA	-4.16* (-7.77, -0.56)	1,157 (180)
**PM**_**2.5**_			
	Total steps of daily walk	-361.85*** (-516.53, -207.16)	1,157 (180)
	Total minutes of daily light physical activity	-8.97*** (-12.28, -5.66)	1,157 (180)
	Total minutes of daily moderate physical activity	-3.73*** (-5.46, -2.01)	1,157 (180)
	Total minutes of daily MVPA	-3.79*** (-5.59, -1.98)	1,157 (180)
**PM**_**10**_			
	Total steps of daily walk	-45.68 (-190.91, 99.54)	1,157 (180)
	Total minutes of daily light physical activity	-3.70* (-6.81, -0.59)	1,157 (180)
	Total minutes of daily moderate physical activity	-0.50 (-2.11, 1.11)	1,157 (180)
	Total minutes of daily MVPA	-0.48 (-2.17, 1.21)	1,157 (180)

*Notes*: Individual fixed-effect regressions were separately undertaken to assess the impact of air pollution concentrations within samples stratified. Models adjusted for all time-varying participants characteristics as displayed in Table 1 (i.e., age, weight), participant-specific temporal order and environmental variables detailed in Table 3 (daily average temperature and rainy day). *P < 0.05; **P < 0.01; ***P < 0.001

Total steps of daily walk demonstrated a significant negative association with AQI. A one-level increase in AQI corresponded to a decrease of 550.04 steps (95% [CI] = -858.97, -241.10; *p* < 0.001). Similarly, for all participants, AQI showed a significant negative association with total daily minutes of LPA, MPA, and MVPA. A one-level increase in AQI was noticed with a decline in LPA, MPA and MVPA by 10.43 min (95% [CI] = -17.07, -3.79; *p* < 0.001), 4.03 min (95% [CI] = -7.48, -0.59; *p* < 0.001) and 4.16 min (95% [CI] = -7.77, -0.56; *p* < 0.001), respectively.

PM_2.5_ demonstrated a significant negative association with total daily walking steps, LPA, MPA and MVPA among total participants. Notably, a one-level increase in PM_2.5_ was observed with a decrease of daily walking steps, LPA, MPA and MVPA by 361.85steps (95% [CI] = -516.53, -207.16; *p* < 0.001), 8.97 min (95% [CI] = -12.28, -5.66; *p* < 0.001), 3.73 min (95% [CI] = -5.46, -2.01; *p* < 0.001,) and 3.79 min (95% [CI] = -5.59, -1.98; *p* < 0.001), respectively.

PM_10_ displayed a significant negative association with total minutes of daily light physical activity, with a one-level increase in PM_10_, corresponding to a decline of 3.7 min in LPA (95% [CI] = -6.81, -0.59; *p* < 0.05). However, no significant negatively association with daily walking steps, MPA and MVPA (*p* > 0.05) was noticed.

## Discussion

This study examined the impact of air pollution levels on physical activity among older adults in China using objective measurements. Additionally, this study is notable for being the first longitudinal study to employ objective physical activity measurements in older adults to estimate relationship between air pollution and physical activity. Moreover, it was the pioneering study to explore whether a relationship existed between AQI and physical activity among older adults.

We found that AQI, PM_2.5_ and PM_10_ were related to decreased physical activity in older adults. AQI demonstrated a strong negative association with daily walking steps, daily LPA, MPA and MVPA among all participants. This study stands as a pioneering contribution to the existing literature on elderly populations, marking the first utilization of the AQI indicator for exploring whether a relationship existed between air pollution and physical activity. Our findings align with earlier research on non-elderly age groups [39, 45–47]. A study on Chinese middle-aged populations showed that a one-unit increase in AQI led to a 20% decrease in physical inactivity [45]. In a longitudinal cohort study encompassing adults aged 18 years and older in California, a statistically substantial decline in accelerometer-based daily walking steps was observed with poor air quality, resulting in an 18% decrease in step counts when the AQI exceeded 200 in comparison to levels below 100 [39]. Furthermore, a cluster randomized controlled trial among Australian children showed that AQI was significantly negatively associated with physical activity, with an increase in AQI by one SD linked to a decline in MPA, VPA and MVPA by 0.86, 0.35 and 1.21 min per day, respectively [47]. It is worth noting that the AQI is defined and measured differently in different countries, making comparisons challenging. Therefore, future studies should consider conducting further research in this area.

Similar to AQI, PM_2.5_ was noticed to have a significantly negatively association with physical activity. A one-level increase in PM_2.5_ led to reduced daily walking steps, LPA, MPA and MVPA among all participants, resulting in declines of 361.85 steps, 8.97 min, 3.73 and 3.79 min per week, respectively. This association aligned with findings from subjectively measured studies undertaken in China and other countries. For example, a study reported that elderly residents in cities with PM_2.5_ higher concentration exhibited elevated levels of physical inactivity [48]. Our longitudinal study conducted among Beijing retirees yielded consistent conclusions [34]. Furthermore, a U.S. cross-sectional study indicated that each PM_2.5_ exposure class increase corresponded to a statistically substantial 16 to 35% increase in probability of physical inactivity [32]. Another cross-sectional study of elderly individuals in the United States found that for each 10-unit (µg/m^3^) increase in PM_2.5_ concentration, the odds of physical inactivity increased by 16% [33]. Furthermore, consistent conclusions have also been reached in studies involving other age groups. A longitudinal study of Chinese freshmen in our previous study demonstrated a reverse correlation between PM_2.5_ level and walking time and VPA [35]. Another cross-sectional study among 18–76 years of age adults also demonstrated a negative correlation between PM_2.5_ concentration and outdoor physical activities [49]. However, the study among adults revealed that PM_2.5_ exerted no effects on the average hourly physical activity, which was inconsistent with our findings [50]. One possible explanation for this inconsistency is that the ages of the participants differed from those in our study.

However, there is no significant relationship between PM_10_ and walking steps, MPA, or MVPA. This result may indicate that the impact of different air quality indicators on physical activity may vary: LPA is associated with AQI, PM_10_, and PM_2.5_, while walking steps, MPA and VPA are only linked to AQI and PM_2.5_. This finding contradicts previous results indicating a direct relationship between an increase in PM_10_ and increased odds of physical inactivity. A U.S. cross-sectional study demonstrated that an increasing concentration of PM_10_ led to heightened odds of physical inactivity [32]. A merger of data from South Korea study showed that walking time decreased by 0.22 h as PM_10_ increased [37]. A cohort study from London in patients with COPD reported that PM_10_ significantly independently associated with reduced pedometer-based daily step counts [42]. The inconsistent of results across studies may be due to factors such as the use of self-reported data in cross-sectional studies [32, 51] or study design related to short-term and long-term pollution exposures [31]. At the same time, a cross-sectional study conducted in the United States suggested that ozone, like PM_2.5_ and PM_10_, is one of the factors affecting physical activity [32], and future research should consider including this variable. This study is preliminary and replication among older adults is warranted.

Some studies advocate that physical activity can still be carried out in air polluted environments. Several health assessments support this viewpoint, indicating that the advantages of physical activity outweigh potential risks linked to air pollution [52, 53]. Public health modeling has also contributed to this perspective by examining the balance of risks and benefits between physical activity and exposure to polluted environments [54]. On the other hand, a minority of studies failed to provide evidence that air pollution exposure offsets the positive effects of physical activities [55–57]. In summary, the comprehensive effects of participating in physical activity in an air-polluted environment remain unclear. Future research should prioritize an examination of their risk-benefit relationship. This study has several strengths. First, we employ accelerometers to objectively measure physical activity of older adults. Many prior investigations into the correlation between air pollution and physical activity relied on subjective measurement methods, potentially introducing biases. As far as we know, this is the pioneering study employing objective physical activity data in delving into the connection of air pollution and physical activity among China’s older adults. Second, our study adopts a longitudinal design, employing an individual fixed effects model during data analysis. This approach effectively mitigates biases that can persist within participants over time. Third, our findings reveal a significant negative correlation between AQI and physical activity within the elderly population. Nevertheless, this study is not without its limitations. Firstly, we were unable to distinguish between outdoor and indoor physical activities, which could introduce variability in air pollution exposure. Secondly, it is worth noting that air pollution exposure may contribute to lagged or cumulative effects, which our study did not account for. Thirdly, our participants were recruited through convenience sampling, potentially not fully representative of all elderly individuals. Hence, further investigations are warranted to address these limitations with other information such as education and socio-economic status.

## Conclusions

This study examined how air pollution is related to base-accelerometer physical activity among older adults. AQI and PM_2.5_ demonstrate an inverse association with physical activity levels among older adults, potentially discouraging their engagement in physical activity. Different air quality indicators may affect physical activity differently. Future studies are warranted to enhance policy interventions aimed at reducing air pollution and promoting physical activity.

## Data Availability

The datasets generated and/or analyzed during the current study are not publicly available due to confidentially reason, but are available from the corresponding author on reasonable request.
